# Backtracked analysis of preleukemic fusion genes and DNA repair foci in umbilical cord blood of children with acute leukemia

**DOI:** 10.18632/oncotarget.24976

**Published:** 2018-04-10

**Authors:** Milan Škorvaga, Matúš Durdík, Pavol Košík, Eva Marková, Marek Holop, Miroslav Kubeš, Judita Puškáčová, Alexandra Kolenová, Igor Belyaev

**Affiliations:** ^1^ Cancer Research Institute, Biomedical Research Center, Slovak Academy of Sciences, Bratislava, Slovak Republic; ^2^ Eurocord-Slovakia, Bratislava, Slovak Republic; ^3^ Children’s Hematology and Oncology Clinic, Faculty of Medicine, Comenius University, Bratislava, Slovak Republic

**Keywords:** leukemia, umbilical cord blood, preleukemic fusion genes, imaging flow cytometry, translocations

## Abstract

The first event in origination of many childhood leukemias is a specific preleukemic fusion gene (PFG) that arises, often in utero, in hematopoietic stem/progenitor cells (HSPC) from misrepaired DNA double strand break (DSB). An immanently elevated level of DSB and impaired apoptosis may contribute to origination and persistence of PFG and donor cell-derived leukemia in recipients of allogeneic transplantation of umbilical cord blood (UCB). We investigated DSB, apoptosis and PFG in the backtracked UCB cells of leukemic patients. RNA from UCB of three patients with acute lymphoblastic leukemia, patient with acute megakaryoblastic leukemia and Down syndrome, and four healthy children was screened for common PFG by RT-qPCR. Presence of PFG was validated by sequencing. Endogenous γH2AX and 53BP1 DNA repair foci, cell populations, and apoptosis were analyzed in UCB CD34+/- cells with imaging and standard flow cytometry. We found *MLL*_2_*-AF4* and *BCR-ABL (p190)* fusion genes in UCB of two out from four pediatric patients, apparently not detected at diagnosis, while UCB cells of *TEL-AML1+* ALL patient were tested negative for this PFG and no PFG were detected in UCB cells of healthy children. No significant difference in DNA damage and apoptosis between UCB CD34+/- cells from healthy children and leukemic patients was observed, while Down syndrome trisomy increased DNA damage and resulted in distribution of cell populations resembling transient abnormal myelopoiesis. Our findings indicate increased genetic instability in UCB HSPC of leukemic patients and may be potentially used for diagnostics and exclusion of possibly affected UCB from transplantation.

## INTRODUCTION

There is compelling evidence that several chromosomal translocations and corresponding preleukemic fusion genes (PFG) such as *MLL-AF4*, *TEL-AML1*, and *AML1-ETO*, which are associated with acute pediatric leukemia, often originate prenatally [1, 2]. Most of the supporting studies report the presence of specific chromosomal translocations in limited proportion of studied cases. For example, Wiemels et al. were able to detect the t(12;21) chromosomal translocation resulting into TEL-AML1 in 9/12 neonatal blood spots (Guthrie cards) from children with *TEL-AML1*^+^ B-cell precursor acute lymphoblastic leukemia (B-ALL) [3]. Other reports show similar results in terms of limited positivity, e.g. 3/9 for *TEL-AML1* [4], or 5/10 for t(8;21)/*AML1-ETO* in Guthrie cards from children with acute myeloblastic leukemia (AML) [5]. These negative neonatal blood spots could either indicate a postnatal origin of the translocation or more probably its prenatal origin that could not be detected due to technical shortcomings of blood-spot screening. Nevertheless, available studies indicate that about 50% of *TEL-AML1* (in ALL) and *AML1-ETO* (in AML) may originate *in utero*. However, there seem to be a few exemptions representing two opposite extremes from this concept. At the one end, it is the *MLL-AF4* fusion gene in t(4;11)^+^ infant leukemia representing an initiating oncogenic hit that obviously arises prenatally during embryonic/fetal development [2]. At the other end, there are (i) the *E2A-PBX1* PFG generated by the t(1;19) chromosome translocation, which might arise in most cases postnatally in B-cell precursors as most neonatal blood spots were tested negative for *E2A-PBX1* sequences [6], and (ii) the T-cell precursor ALL initiating oncogenic mutations and clone-specific molecular markers seem to be generated after birth as suggested by a study in which the positivity was found only in 1/16 of Guthrie cards, despite using PCR approaches with a sensitivity level up to 10^-5^ [7]. A recent analysis of Guthrie cards from *TEL-AML1*^+^ leukemic patients showed that all retrospective blood spots screened by either R-T qPCR (0/15) or nested PCR (0/8), which both allowed detection of one TEL-AML1^+^ cell *per* 10^5^ cells, were tested negative for the corresponding fusion transcripts [8]. The yield and quality of total RNA based on the analysis of control human b-glucuronidase gene suggested that the level of *TEL-AML1* PFG isolated from Guthrie cards may be below 1 × 10^-5^.

Umbilical cord blood (UCB) represents an alternative source that allows the backtracked analysis of potentially prenatal PFG. The incidence of *TEL-AML1* in UCB of healthy neonates is apparently controversial: most studies have proposed a frequency of about 1% [9–12], while the other found a much lower incidence, 0.01% [13, 14]. Since Guthrie cards rarely contain more than 10^5^ cells [13], UCB screening is preferable to neonatal blood spots analysis in identification of children at risk of developing PFG^+^ leukemia. As of December 1^st^ 2014, more than 4 million UCB units have been stored worldwide, see review [15]. In general, UCB possess several advantages over bone marrow (BM), including abundant and immediate availability, easy collection, risk-free donation, reduced risk of blood-borne infection and graft-versus-host disease. In comparison with adult BM, UCB cells are less mature, have longer telomeres and greater proliferative potential. In pediatric ALL, hematopoietic stem cell transplantation (HSCT) is commonly considered for children with extremely high risk features, e.g. hypodiploidy, induction failure [16]. Due to the relatively poor efficiency of chemotherapy treatment of pediatric AML, allogeneic HSCT has commonly been used for children in remission [17]. In addition, allogeneic HSCT is the only curative treatment for rare pediatric leukemias, such as chronic myeloid leukemia, juvenile myelomonocytic leukemia, and myelodysplastic syndromes (MDS) [16]. Autologous UCB transplantation has been predominantly used for brain injuries (82%) [18]. In addition, its curative effects have been manifested most in children with hemoglobinopathies and inherited metabolic diseases such as Hurler syndrome, Krabbe syndrome, Metachromatic leukodystrophy [15].

Donor cell leukemia (DCL) is a rare but well-recognized complication that occurs after allogeneic transplantation [19]. Although the incidence of DCL is very rare, its prognosis is extremely poor. The DCL mortality remains very high [20]. One possible mechanism for the development of DCL is that preleukemic clone was already present in the donor before transplant, but had remained undiagnosed. Thus, the screening of UCB for preleukemic clones based on analysis of PFG may be of high importance for preventing DCL and evaluation of groups at increased risk for leukemia.

We and other groups have shown that the incidence of PFG in UCB cells may be relatively high and part of them may not be relevant to diagnosis [21]. Importantly, all PFG, regardless time of their origination, require DNA double strand break (DSB) as a prerequisite for the formation of chromosomal translocations (and hence the fusion genes). Endogenous DSB are primarily occurring due to oxidative DNA damage. There is evidence that DSB correlate with presence of *BCR-ABL* and *TEL-AML*1 suggesting possible link between increased endogenous damage and risk of leukemia [22].

The major objective of our study was to investigate whether UCB cells of selected pediatric patients with acute leukemia had an impaired DNA damage response and whether they contain PFG non-related to diagnosis. Accordingly, we have performed the analysis of DNA repair foci in cells obtained from UCB of patients and healthy subjects. The rationale for the use of this methodological approach is that monitoring DNA repair foci is the most sensitive and specific method for measuring DSB [23, 24]. These foci are formed at the site of DSB as a result of the recruitment of DNA damage proteins and usually monitored using antibodies to γH2AX and 53BP1 proteins [25]. Because hematopoietic stem/progenitor cells (HSPC) are considered to be the main target for origination of leukemia and apoptosis is the key process for eliminating damaged cells, we analyzed DSB and apoptosis in CD34+ HSPC in comparison to CD34- lymphocytes. In addition, the patient’s UCB cells were subjected to an R-T qPCR analysis with the sensitivity 1 – 3 × 10^-5^ for the presence of the most prevalent PFG, namely: TEL-AML1, *MLL-AF4, BCR-ABL* (p190). Due to limited amount of total RNA, four matched control UCB from healthy subjects were screened for the two PFG found in patient’s cord blood, i.e. *MLL-AF4* and *BCR-ABL* (p190). An accurate screening of UCB or BM for PFG may have also a clinical significance, since PFG-containing UCB/BM used for allogeneic HSCT may cause a DCL in recipient.

Basic information on probands, including diagnosis and PFG status, age at diagnosis in patients, and sex of all enrolled probands are provided in Table 1. More information on patients, chemicals and methods used in this work are included in Materials and Methods.

**Table 1:** Proband information: diagnosis and PFG status, age at diagnosis in patients, and sex

Proband No.	Diagnosis, PFG	Age at diagnosis/sex
1	**ALL**, PFG^-^	2-year/girl
2	**AML/DS**, PFG^-^	1.4-year/boy
3	**ALL**, TEL-AML1^+^	3-year/boy
4	**ALL**, *AML1* gene duplication	5.4-year/boy
5	healthy	boy
6	healthy	boy
7	healthy	girl
8	healthy	boy

## RESULTS

In attempt to increase the chance of detecting rare PFG in UCB samples, we increased the amount of RNA in reverse transcription reaction from a standard 1 μg to 2 μg and 3 μg, when possible. Our unpublished data indicated an inhibition of RT reaction with further increase of total RNA till 5 μg. The results of R-T qPCR analysis for the presence of common PFG along with *c-Abl* housekeeping gene analysis are shown in Table 2.

**Table 2:** R-T qPCR screening of patient’s cord blood and validation by sequencing

UCB bag #	Total RNA	*c-Abl* C_t_(per reaction copy number)	*TEL-AML1*	*MLL*_*1*_*-AF4*	*MLL*_*2*_*-AF4*	*BCR-ABL* p190
1a	2μg	**24.82** (34,880)	0/1	0/1	0/1	0/1
	3μg	**24.42 (**45,940)	0/1	0/1	0/1	1/1^**^ 40.75 (2.363)
1b	2μg	**25.35** (24,000)	0/1	1/1^**^ 38.59 (9.468)	0/1	0/1
	3μg	**24.81** (34,900)	0/1	0/1	0/1	0/1
	Summary	∼ 14,000 *per* 10^5^ cells	negative	negative	negative	negative
2	2μg	**23.45 (**90,240)	0/1	0/1	1/1^*^ 37.67 (1.949)	1/1^*^ 40.43 (2.866)
	3μg	**23.02 (**121,900)	0/2	0/2	0/2	2/2^*^ 40.46 (2.807) **39.29** (5.684)
	Summary	∼ 42,000 *per* 10^5^ cells	negative	negative	**positive**	**positive**
3	2μg	**25.66** (19,280)	0/1	0/1	0/1	1/1^**^ 40.53 (2.690)
	3μg	**25.22** (26,270)	0/2	0/2	0/2	2/2^*^ 40.22 (3.274) **40.35** (3.001)
	Summary	∼ 9,000 *per* 10^5^ cells	negative	negative	negative	**positive**
4	2μg	**25.32 (**12,110)	0/3	3/3^**^ 35.88 (6.156) **35.89** (6.134) **34.06** (10.700)	1/3^**^ 37.45 (2.686)	0/3
	3μg	**24.11 (**26,860)	0/3	2/3^**^ 34.12 (20.160) **35.24** (9.476)	0/3	0/3
	Summary	∼ 7,500 *per* 10^5^ cells	negative	negative	negative	negative

Data show the quality of isolated total RNA as C_t_ and copy number of *c-Abl* control gene, and test results for *TEL-AML1*, *MLL-AF4*, and *BCR-ABL* (*p190*) fusion transcripts. In samples tested PFG positive with R-T qPCR, C_t_ and copy number of PFG are shown. Finally, only samples with fusions genes confirmed by sequencing were considered as positive.

^*^ - confirmed by sequencing, ^**^ - not confirmed by sequencing.

UCB cells of patient #1 diagnosed with B-ALL were analyzed from two collection bags, #1a and #1b. Samples #1a and #1b were initially tested positive for *BCR-ABL* (p190) and *MLL*_*1*_*-AF4*, respectively. However, this positivity could not be confirmed by sequencing.

Patient #2 was diagnosed with AML, subtype acute megakaryoblastic leukemia and Down syndrome (AML/DS). This patient’s UCB mononuclear cells (MNC) exhibited the best parameters of isolated RNA from all screened samples, reaching > 40,000 copies of *c-Abl* control gene *per* 100,000 cells in average. The patient’s MNC were tested positive for both *MLL*_*2*_*-AF4* and *BCR-ABL* (p190) as defined by R-T qPCR and confirmed by sequencing (Supplementary Sequencing analysis).

UCB MNC of patient #3 represented the only patient’s sample with a chromosomal translocation, namely t(12;21)/*TEL-AML1* that was detected at diagnosis. However, the patient’s UCB was found negative for the *TEL-AML1.* Interestingly, UCB cells of this patient were tested positive for *BCR-ABL* (p190) as validated by sequencing.

Cells of ALL patient #4 contained *AML1* gene duplication at diagnosis. Initially, the UCB cells of this patient were found positive for *MLL*_*1*_*-AF4* PFG (5/6), and *MLL*_*2*_*-AF4* (1/6), however, the sequencing could not confirm this positivity.

Since the patient’s UCB MNC were tested positive for *MLL*_*2*_*-AF4* and/or for *BCR-ABL (p190)* and their positivity was validated by sequencing, the control UCB MNC from healthy subjects were screened for the presence of the same PFG. All control UCB MNC were tested negative for both the *MLL*_*2*_*-AF4* and *BCR-ABL* (p190) PFG (Supplementary Table 1).

**Figure 1:**
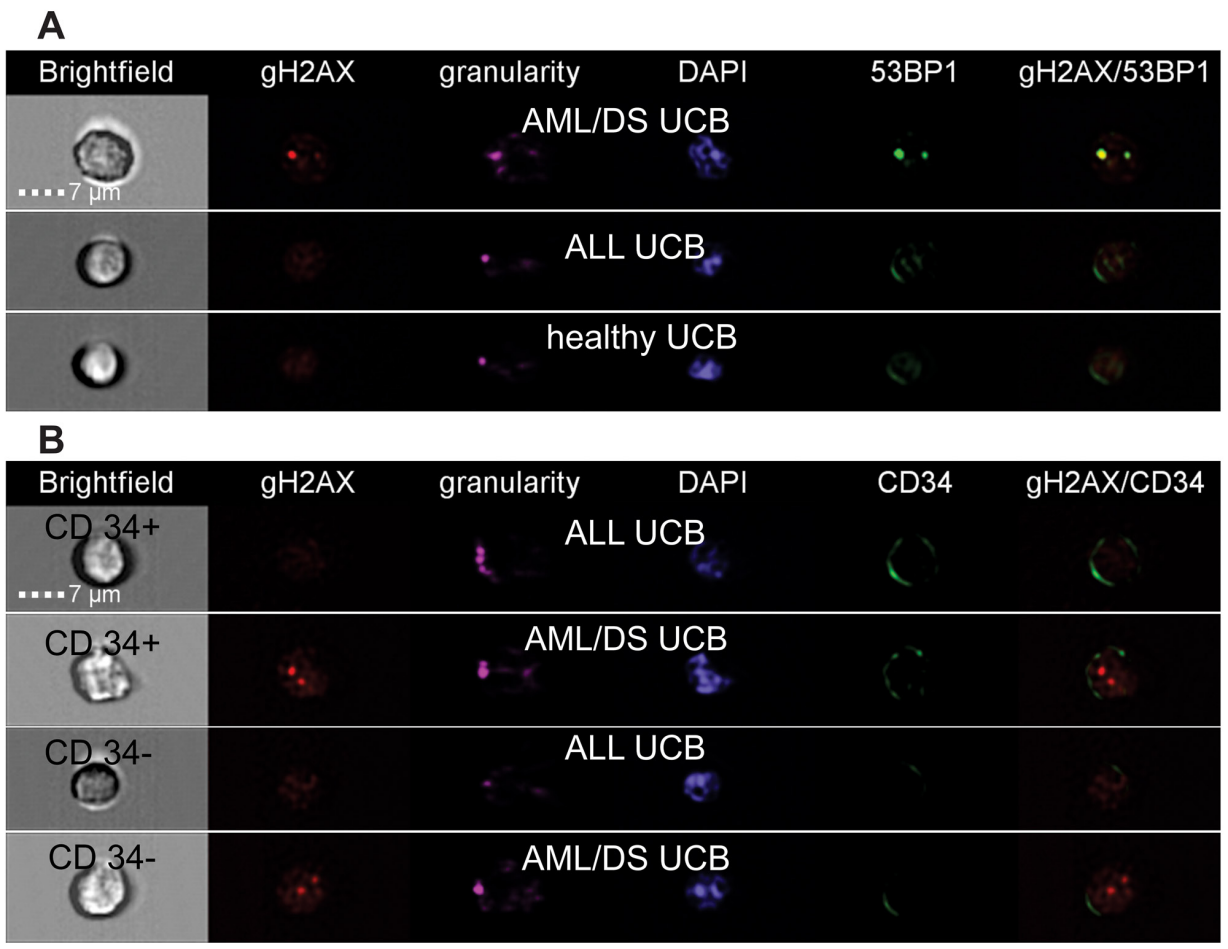
Representative images of cells from healthy and patient’s UCB obtained by imaging flow cytometry MNC were stained with either monoclonal γH2AX or polyclonal 53BP1 antibodies **(A)** or polyclonal γH2AX and monoclonal CD34-APC conjugated antibodies **(B)**. Panel A shows representative images of UCB cells from patient AML/DS #2, ALL patient #4, and healthy subject #6. Panel B shows representative images of the CD34+ and CD34- UCB cells from patient AML/DS #2 and ALL patient #4.

**Figure 2:**
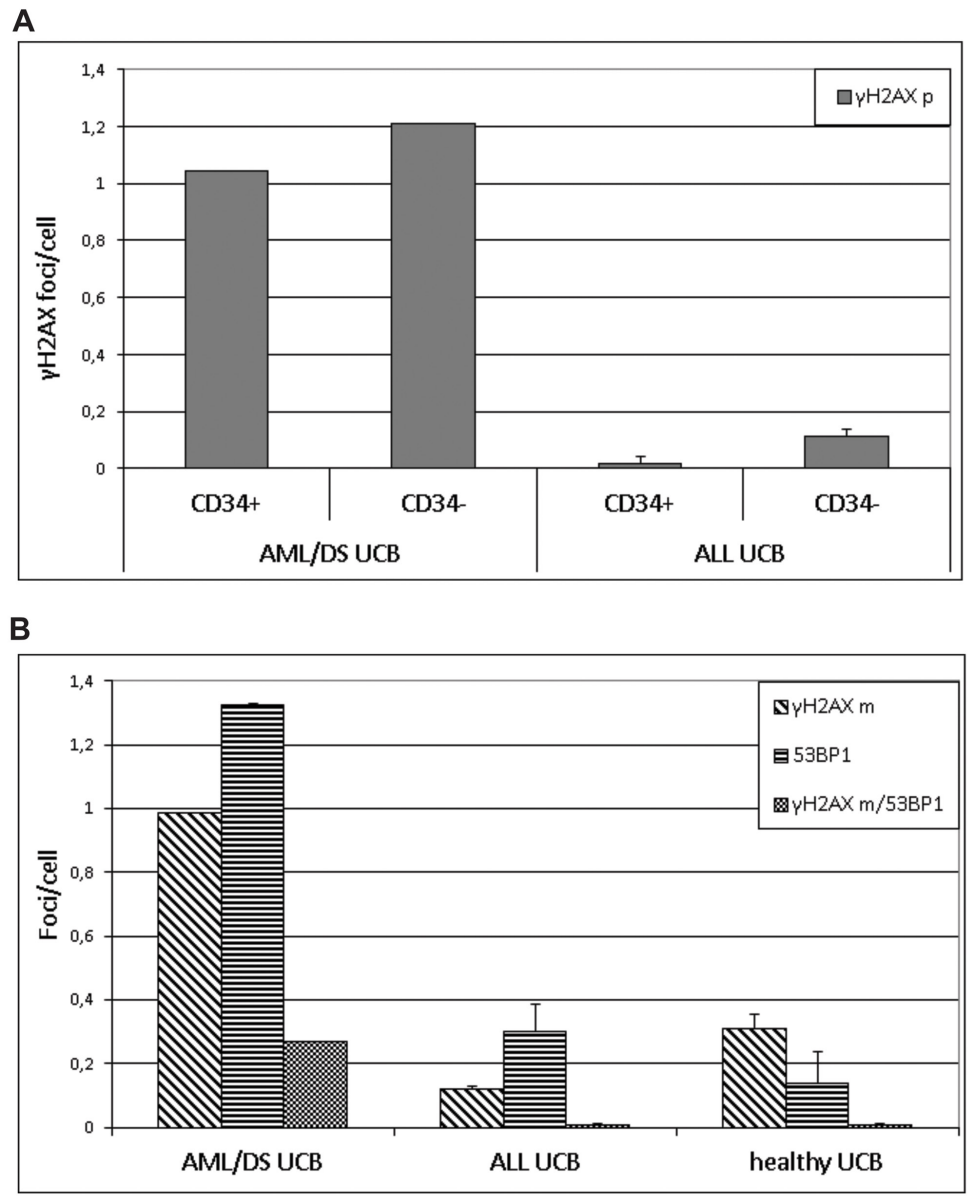
DNA repair foci The level of γH2AX foci in CD34+ HSPC and CD34- lymphocytes stained by polyclonal γH2AX antibody (γH2AXp) in combination with CD34-APC conjugated antibody **(A)** and level of γH2AX/53BP1 stained by monoclonal γH2AX antibody (γH2AXm) in combination with polyclonal 53BP1 as analyzed in UCB cells from patient AML/DS #2, three ALL patients, and healthy subjects **(B)**.

DSB were analyzed by imaging flow cytometry using antibodies to 53BP1 and γH2AX proteins (Figure 1). In line with previous publications [25], 53BP1 and γH2AX did not 100% co-localize due to different kinetic properties of these proteins in assembling and disassembling of DNA repair foci at the location of DSB (Figure 2B). Analysis of DNA repair foci with monoclonal γH2AX (γH2AXm) antibody in combination with polyclonal 53BP1 revealed similar DNA damage in UCB cells of healthy subjects and pediatric ALL patients while DSB levels were strikingly higher in UCB cells from child diagnosed with AML/DS (Figure 2B). These data were confirmed by analyzing DNA repair foci with polyclonal γH2AX (γH2AXp) antibody (Figure 2A). Both CD34+ and CD34- cells from UCB of the AML/DS patient with chromosome 21 trisomy also showed an elevated level of the γH2AXp foci (Figure 2A). Due to insufficient cell number, CD34+ cells were not analyzed in the UCB samples of healthy subjects. However, the amount of γH2AXp foci seen in patient`s CD34+ UCB cells was consistent with our previously published data on CD34+ cells from UCB of healthy newborns [26]. In general, the same levels of endogenous foci were found in the UCB MNC of children that later develop leukemia (except patient with AML/DS) and those of healthy probands regardless the type of analysis: 53BP1, γH2AXp, or γH2AXm/53BP1 foci (ANOVA, p = 0.12).

In line with our previous finding with UCB MNC from PFG^+/-^ healthy neonates [27] we found a significantly decreased level of the γH2AX foci in CD34+ cells from the patients’ cord blood MNC in comparison to CD34- cell populations (*t*-test, p = 0.013) (Figure 2A).

**Figure 3:**
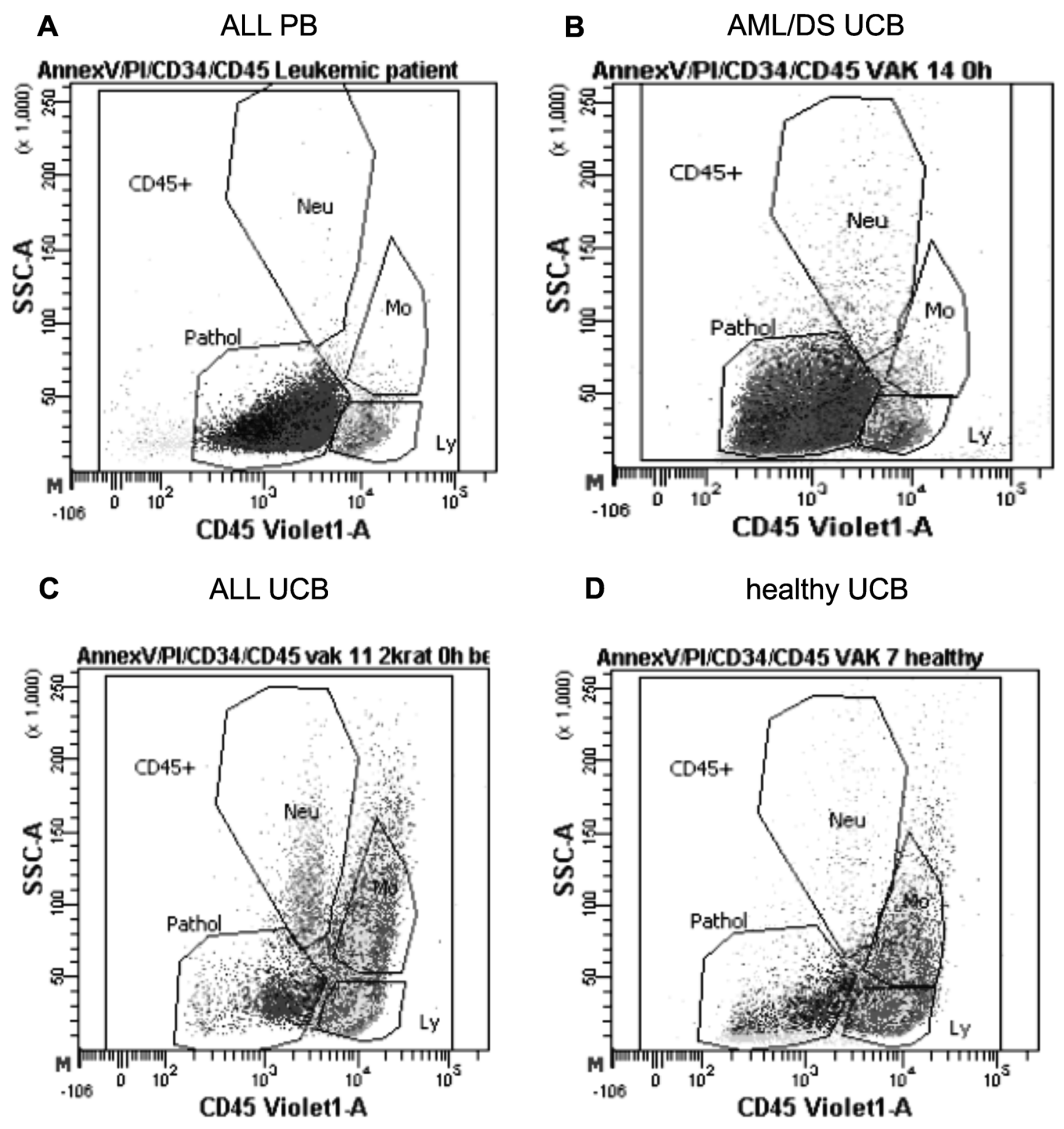
Distribution of pathological and normal cells Representative distributions of pathological (Pathol) and normal cell populations (lymphocytes (Ly), monocytes (Mo) and neutrophiles (Neu)) gated according to the CD45 marker and side scatter obtained by flow cytometry in: **(A)** peripheral blood (PB) of ALL patient #4, **(B)** UCB of AML/DS patient #2, **(C)** UCB of ALL patient #4 and **(D)** UCB of healthy subject #2.

A new interesting finding of this study followed from flow cytometry analysis of AML/DS patient’s UCB MNC that revealed that majority (≥ 80%) of MNC fitted to the gate routinely used for pathological cells at diagnosis [28]. Figure 3 illustrates this finding by comparing distributions of gated pathological and normal MNC populations in ALL patients’ peripheral blood, ALL UCB, AML/DS UCB, and healthy UCB. This type of cell distribution was not seen in the UCB samples from other patients and healthy subjects suggesting that combination of chromosome 21 trisomy with predisposition to leukemia may solely be responsible for pathology-like gating of the UCB cells.

The rate of apoptosis was the same in cells from healthy controls and patients as measured both in CD34- lymphocytes (p = 0.24) and CD34+ HSPC (p = 0.98) However, taking both groups together, we observed higher level of live cells in HSPC compared to lymphocytes (p = 0.036) and lower level of early apoptotic cells in HSPC (CD34+) population compared with lymphocytes (p = 0.017). This finding confirmed our previous data showing increased resistance of human HSPC [26].

## DISCUSSION

Few reports have shown that neonatal blood spots (also known as Guthrie cards) of substantial proportion of PFG^+^ pediatric ALL/AML patients, in which a specific chromosomal translocation/fusion gene was detected at diagnosis, were tested negative for the corresponding preleukemic fusion gene [3, 29]. The major advantage of our present study consists of using UCB MNC for PFG screening and examining cDNA. The drawback of our approach is the use of frozen UCB from standard collection bags, thus not eliminating a possible degradation of PFG potentially present in the analyzed samples. Another important question is whether widely used R-T qPCR methodological approach for PFG screening of MNC is adequately sensitive and specific, because precise and unambiguous determination of positivity of UCB samples has an important clinical aspect. The presence of preleukemic clone(s) in the donor UCB at the time of allogeneic cord blood transplantation may represent „the 1st hit“ to the recipient and subsequently, additional hit(s) may lead to DCL in the recipient. Although DCL is very rare and its incidence was reported to be from 0.13% to 1.2% [30–32] or even up to 5% [33], the DCL patients have extremely poor prognosis, reaching median survival only 6 months [20].

This study included MNC isolated from UCB of four children with acute leukemia (three with ALL, one with AML), one of them being tested positive for *TEL-AML1* fusion gene at diagnosis. Overall, this work brought several interesting results as discussed below.

We failed to identify *TEL-AML1* in UCB from the patient #3 diagnosed with *TEL-AML1*^*+*^ pediatric ALL. Quantification of c-*Abl* control gene reaching 9,000 copies *per* 100,000 cells in average (Table 2) suggested that isolated total RNA was suitable for R-T qPCR since (i) we were able to detect other type of PFG *(BCR-ABL p190)* in this sample, and (ii) in previous study, PFG positivity was found and confirmed by sequencing even in samples with ∼ 3,000 copies of control gene *per* 100,000 cells [27]. Increase of the standard 1 μg amount of the total RNA used for cDNA synthesis resulted in an enhancement of c-*ABL* copy number. However, it did not change the negative outcome for *TEL-AML1*. In comparison, in our previous study we confirmed by sequencing 5 out of 5 UCB samples tested positive for TEL-AML1 among 500 Slovak newborns screened by the identical methods [27]. The crucial difference is that frozen UCB samples were processed in this work while fresh UCB were used for cell isolation in the former study. Processing of frozen blood might increase detection threshold due to excessive degradation of PFG. Thus, we may assume that *TEL-AML1* fusion gene might arise in ALL patient #3 *in utero* producing extremely low amount of *TEL-AML1+* cells, which were under increased detection threshold for frozen blood. However, we cannot rule out a possibility that the *TEL-AML1* fusion gene in ALL patient #3 might arise postnatally. Essentially, the failure to identify *TEL-AML1* in the UCB MNC of pediatric patient with *TEL-AML1*^*+*^ ALL at diagnosis is in line with estimated ∼ 50% positivity of Guthrie cards for the initiating PFG observed in previous studies [4, 34].

For the first time, we identified in patient’s UCB samples and confirmed by sequencing fusion transcripts (*BCR-ABL* and *MLL*_*2*_*-AF4),* which were not related to diagnosis (Table 2). Importantly, their incidence considerably exceeded incidence of *BCR-ABL* and *MLL*_*2*_*-AF4* detected in UCB of healthy children both in this work and in our previous study on 500 healthy newborns (Table 3). It should be noted that some studies reported relatively high incidence of PFG in healthy subjects [21], e.g. a report showing ∼ 40% (21 of 50) of UCB samples and ∼ 74% (59/80) of peripheral blood of healthy individuals tested positive for *BCR-ABL* (p190) [35]. However, these higher values were not further validated by additional analysis to exclude false positivity. Thus, the obtained data may suggest increased genetic instability in hematopoietic cells of children long before overt leukemia has been diagnosed. In order to test this option, UCB MNC from patients and healthy individuals were subjected to DNA repair foci analysis. The level of endogenous γH2AX/53BP1 foci in UCB cells of healthy subjects was comparable with our previously published data [26, 27, 24]. Our data showed no significant differences in DNA damage between patient’s and healthy UCB MNC, most probably due to an extremely low proportion of PFG^+^ clones, which are presumably characterized by genetic instability, in patient’s samples. However, DNA damage in UCB cells of the AML/DS pediatric patient #2 was strikingly higher than in all other samples (Figures 1 and 2). Significantly increased levels of DNA repair foci observed in UCB CD34+/CD34- cells from child with AML/DS are consistent with reports showing an elevated generation of oxygen radicals in DS cells [36–38]. Given that there was no difference in endogenous DNA damage between CD34+/CD34- cells from PFG positive and PFG negative UCB [27], these results seem to be unrelated to the presence of preleukemic fusion genes, but rather primarily caused by chromosome 21 trisomy as suggested by previously published data [39]. In contrary to all other samples where either one or none PFG was found, two preleukemic fusion genes, *BCR-ABL (p190)* and *MLL*_*2*_*-*AF4, were detected in UCB cells from the AML/DS pediatric patient #2. This fact is in line with higher DNA damage in cells of AML/DS pediatric patient revealed by the DNA repair foci assay and increased risk for leukemia in children with DS [40–42].

**Table 3:** Incidence of common ALL-associated PFG in (i) UCB of healthy Slovak neonates, (ii) backtracked UCB of healthy children, and (iii) backtracked UCB of leukemic pediatric patients

	500 UCB healthy neonates	4 UCB healthy children	4 UCB leukemic children
**Incidence** (*BCR-ABL*, *MLL-AF4*)	0.8 – 5%	0%	25 - 50%
**Reference**	Kosik *et al* (2017)	This study	This study

We have shown, for the first time, that majority of UCB MNC from the AML/DS pediatric patient fitted to the gate routinely used for pathological cells at diagnosis strongly resembling overt leukemia (Figure 3). This phenomenon is called transient abnormal myelopoiesis (TAM) and seen in blood and bone marrow of children with DS at or soon after birth [43]. Approximately 20% of patients with TAM develop clonally related overt leukemia within the first 4 years of life [43]. In addition, TAM can occur in phenotypically normal neonates and in these cases infants are either mosaic for trisomy 21 or have an acquired trisomy 21 restricted to the leukemic clone [44]. In both cases, the neonates with TAM have similar clinical and biological features to TAM occurring in DS patients. These children without DS show the same increased risk for AML, about 20%, as in DS patients with TAM. Our finding of TAM in UCB of AML/DS patient, along with established higher endogenous DNA damage and PFG incidence in UCB of this patient, may provide a new approach for clinical application by screening UCB for TAM, PFG and DSB to the aim of early diagnostics and excluding these UCB with preleukemic disorder from an UCB transplantation bank. However, whether our finding is typical for UCB of leukemic patients with DS remains to be further investigated with a larger group of DS leukemic patients.

In conclusion, we found no difference in the level of endogenous DNA damage and apoptosis between CD34+/CD34- UCB cells of pediatric leukemic patients and healthy subjects. Possible increased DNA damage in specific leukemogenic populations of HSPC from leukemic patients remains to be investigated. We found increased PFG incidence in UCB of leukemic patients possibly indicating increased genetic instability in HSPC and need to screen the fresh UCB samples by implementing new approaches with a higher specificity, thus targeting leukemogenic stem/progenitor cells and excluding cells, which are not capable of initiating and sustaining leukemogenesis. The research effort should be directed towards characterization and definition of specific target cell populations where the preleukemic lesions initially arise. DNA-based methods such as flow fluorescence in situ hybridization (flow-FISH) may be used in an effort to further reduce the level of PFG false positives that might be formed by alternative splicing.

## MATERIALS AND METHODS

### Patients

Group of leukemic patients consisted of three boys and one girl (Table 1). In addition, the Table 1 contains relevant information on four matched by sex healthy children whose UCB cell analyses represent an essential part of the study. Patients were treated at the Department of Pediatric Hematology and Oncology in Bratislava. This study was approved by the local ethics committee. Children’s parents gave written informed consent to participate in the study. Diagnosis of leukemia was based on the French-American-British classification and flow cytometric immunophenotyping using a standard set of monoclonal antibodies according to the European Group for Immunological Characterization of Leukemia [45]. FISH, immunophenotyping and PCR screening for common PFG were routinely performed on samples from each patient. Among four pediatric leukemic patients enrolled to this study three were diagnosed with ALL and one with AML, namely AML M7 or acute megakaryoblastic leukemia, and Down syndrome (DS). Only patient #3 contained a common translocation/PFG, namely ALL-associated t(12;21)(p13;q22)/*TEL-AML1* as revealed by routine screening of bone marrow by FISH at diagnosis and confirmed two and five weeks after beginning the treatment when assessing bone marrow by R-T qPCR.

### Frozen UCB from pediatric patients with acute leukemia and healthy subjects

Our sample set consisted of whole UCB collection bags backtracked from four pediatric ALL/AML-patients and four healthy donors (Table 1). The UCB samples were routinely collected from newborns born after full-term pregnancies by Eurocord-Slovakia, Bratislava, and stored frozen in nitrogen approximately the same time, 1 - 5 years, for both patient’s and control group. Patient #1 and two healthy controls provided two UCB bags each; therefore, we have analyzed cells from five and six UCB bags from groups of patients and healthy controls, respectively. These bags were obtained with parental informed consent by syringing out the placenta through the umbilical cord after the cord has been detached from the newborn.

### Isolation of mononuclear cells from frozen UCB

The total UCB from each collection bag was thawed and then proceeded according to validated UCB washing protocol (Standard operating procedure of Eurocord-Slovakia). Briefly, the UCB in cryobag was thawed in the 37°C water bath, and then 10% solution of Dextran 40 (½ of the UCB volume) was added to the cryobag under constant stirring. Then the content of the cryobag was transferred into the mixing bag and 5% solution of human albumin (½ of the original UCB volume) was added under constant stirring. Diluted UCB was then centrifuged in the mixing bag at 400 × g, 15 min at 20°C. After centrifugation, the supernatant volume of diluted UCB was reduced to original volume in plasma expressor and mononuclear cells (MNC) were isolated from the resuspended cell pellet by gradient centrifugation as previously described [22]. If possible, up to 5 × 10^7^ MNC were used for PFG screening and minimum of 2 × 10^7^ MNC were employed for imaging and standard flow cytometry.

### Isolation of RNA from UCB MNC

Total RNA was usually isolated from 5 × 10^7^ UCB MNC by RNAzol method following manufacturer’s protocol (Molecular Research Center, Cincinnati, USA). Apparently, the amount, concentration and purity of total RNA met standard parameters required for cDNA synthesis and R-T qPCR analysis, i.e. concentration at least 100 ng/μl and λ_260/280_ where 260/280 is subscript as in the Supplementary Table 2 between 1.7 and 2.1 (Supplementary Table 2). The quality of total RNA was examined by analysis of c-Abl housekeeping gene copy number that significantly exceeded 10,000 copies *per* 100,000 cells, which is regarded as a suitable amount for R-T qPCR screening, in bags #1a, 1b, 2 and was slightly lower in bags #3 and 4 (Table 2) with ∼ 9,000 and 7,500 copies, respectively. Other values are provided in the Supplementary Table 2. Obviously, all total RNA samples were of sufficient quality for further analysis.

### Real-time quantitative PCR (R-T qPCR)

cDNA used as a template in the R-T qPCR was obtained by reverse transcription of total RNA in the standard reaction following manufacturer’s protocol (Thermo Scientific, St. Leon-Rot, Germany), as described in detail previously [46]. In attempt to enhance probability of the target sequences to be included in the reaction, we used increased amount of total RNA, 2 and 3 μg per reaction. The TaqMan universal PCR master mix was from Solis BioDyne (Tartu, Estonia). The primers and probes designed by Gabert and colleagues were synthesized by VBC-Biotec (Vienna, Austria) [47]. The plasmid standards with individual fusion genes were from Ipsogen (now Qiagen Marseille, France). The R-T qPCRs were performed on BioRad CFX96 instrument following the standard protocol [47]. RNA from each UCB was analyzed at least in triplicates.

### Sequencing of R-T qPCR products

The qPCR products were first re-amplified in a standard PCR, subcloned into pUC18 vector, and inserted DNA fragment was sequenced in both directions using M13/pUC forward and reverse sequencing primers, respectively. Sequencing was performed using BigDye^®^ Terminator v3.1 Cycle Sequencing Kit, following manufacturer’s protocol (Applied Biosystems, Austin, TX, U.S.A.). Since our previous screening of UCB from 500 probands suggested a high incidence of PFG false positives, the UCB in this study has been considered as positive when the initial PFG positivity observed by R-T qPCR was also confirmed by sequencing of qPCR product.

### Imaging flow cytometry

Cells from patient’s and control UCB collection bags were analyzed for endogenous DSB using imaging flow cytometry (ImageStream, Amnis, Seattle, USA) as previously described [24]. Majority of adherent monocytes were removed by 2 h incubation of MNC in CO_2_ incubator. One MNC aliquot (about 3 × 10^6^ cells) from each UCB bag was probed using polyclonal γH2AX antibody (γH2AXp, Cell Signaling Technology Danvers, MA, USA) in combination with CD34-APC conjugated antibody for HSPC (MACS Miltenyi Biotec, Germany) and another MNC aliquot was analyzed with monoclonal γH2AX antibody (γH2AXm, Novus Biologicals, United Kingdom) in combination with polyclonal 53BP1 antibody (Novus Biologicals, United Kingdom). The number of γH2AX and 53BP1 foci and their co-localization, which is considered to be the most reliable DSB marker, was enumerated in at least 2000 lymphocytes and 25 HSPC per sample.

### Flow cytometry

The viability of cells was analyzed by BD FACSCanto^™^ II cell flow cytometer using propidium iodide Annexin V-FLUOS staining kit (Roche, Switzerland). Lymphocytes, neutrophils, monocytes, pathological cells and HSPC were gated using the fluorescent specific surface markers CD45 (BD Biosciences, San Jose California, USA) and CD34 (MACS Miltenyi Biotec, Bergisch Gladbach, Germany), respectively. In average, HSPC represented 1% of MNC. Both subpopulations were analyzed for % of live, early apoptotic and late apoptotic/necrotic cells. Not less than 50 000 lymphocytes and 500 CD34+ HSPC were taken for analysis.

### Statistics

Statistical analysis was carried out using Statistica 8.0 (Statsoft, Dell software, Round Rock, TX). The Analysis of variance (ANOVA) was used to estimate variances in UCB MNC from patients and control subjects. Comparison between HSPC and lymphocytes was performed with two tailed *t*-test. The results were considered significantly different at p<0.05.

## SUPPLEMENTARY MATERIALS TABLES





## Abbreviations

ALL: acute lymphoblastic leukemia
AML: acute myeloid leukemia
B-ALL: B-cell precursor acute lymphoblastic leukemia
BM: bone marrow
DCL: donor cell-derived leukemia
DS: Down syndrome
DSB: double strand break
FISH: fluorescence in situ hybridization
HSCT: hematopoietic stem cell transplantation
HSPC: hematopoietic stem/progenitor cell
MNC: mononuclear cell
PCR: polymerase chain reaction
PFG: preleukemic fusion gene
R-T qPCR: real-time quantitative polymerase chain reaction
TAM: transient abnormal myelopoiesis
UCB: umbilical cord blood
