# Supplementary material for: Backtracked analysis of preleukemic fusion genes and DNA repair foci in umbilical cord blood of children with acute leukemia
Source: Oncotarget. 2018 Apr 10;9(27):19233–44. doi: 10.18632/oncotarget.24976 (PMC5922391; doi:10.18632/oncotarget.24976)
Supplement: Supplementary file 1 [file oncotarget-09-19233-s001.pdf]

Backtracked analysis of preleukemic fusion genes and DNA repair foci in umbilical cord blood of children with acute leukemia

SUPPLEMENTARY MATERIALS

Supplementary Table 1: Control UCB collection bags from healthy donors were tested negative for MLL2-AF4 and BCR-ABL (p190)

UCB bag #	Total RNA	c-Abl [copies]	MLL_2-AF4	BCR-ABL (p190)
**5**	1μg	38,470	0/3	0/3
	3.2μg	1,00,700	0/3	0/3
**6**	1μg	40,820	0/3	0/3
	2.4μg	78,750	0/3	0/3
**7a**	0.59μg	26,995	n.d.	n.d.
**7b**	1μg	38,590	0/3	0/3
	2μg	38,090	0/3	0/3
**8a**	1μg	9,654	0/3	0/3
**8b**	1.7μg	13,760	0/3	0/3
	**R-T qPCR**	**E; R^2**<br>slope	**99.4%; 0.993**<br>-3.337	**98.5%; 0.994**<br>-3.358

Data include total RNA amounts used for cDNA synthesis, c-Abl control gene copy number, number of positives *per* triplicate and R-T qPCR parameters, including % efficiency (E), R^2 value and slope of standard curve.

Supplementary Table 2: RNA isolation from UCB MNC

UCB bag #	MNC amount	total RNA					
		c [ng/µl]	purity $\lambda_{260/280}$	yield		c-Abl [1µg RNA, cDNA]	
				total, µg	/cell, pg	C_t	copy number
1a	5×10^7	443.7	2.04	88,7	1.77	26.17	13,490*
1b	5×10^7	454.9	2.03	90.9	1.82	26.44	11,180*
2	5×10^7	768	2.04	153.6	3.07	24.43	45,710*
3	3×10^7	575.1	2.03	34.5	1.73	26.51	10,650*
4	5×10^7	1,249.70	2.04	99.9	1.99	23.70[a]	14,780**
5	5×10^7	328.8	1.96	98.6	1.97	24.45	38,470*
6	5×10^7	243.2	2.09	72.9	1.46	24.38	40,820*
7a	n.d.	58.5	1.87	16.09	n.d.	24.95	26,995*
7b	5×10^7	202.4	1.95	55.7	1.11	24.54	38,590*
8a	25×10^6	174.3	1.51	19.5	0.78	27.22	9,654*
8b	5×10^7	106	1.96	31.8	0.64	n.d.	n.d.

Data show parameters of isolated total RNA including concentration, purity, yield, and quality estimated by measurement of *c-Abl* control gene copy number using R-T qPCR. UCB MNC from leukemic patients (bags 1 – 4) and healthy children (bags 5 -8) were analyzed.

* R-T qPCR parameters (**E** = 101.4%; $\mathbf{R^2}$ = 0.999; **slope** = -3.290).

** R-T qPCR parameters (**E** = 97.6%; $\mathbf{R^2}$ = 0.997; **slope** = -3.381).

Supplementary Sequencing analysis.

See Supplementary File 1
