# Supplementary material for: Backtracked analysis of preleukemic fusion genes and DNA repair foci in umbilical cord blood of children with acute leukemia
Source: Oncotarget. 2018 Apr 10;9(27):19233–44. doi: 10.18632/oncotarget.24976 (PMC5922391; doi:10.18632/oncotarget.24976)
Supplement: Supplementary file 2 [file oncotarget-09-19233-s002.doc]

**Supplementary Sequencing analysis**

Gabert’s BCR-ABL1 (p190) sequence:

CTGGCCCAACGATGGCGAGGGCGCCTTCCATGGAGACGCAGAAGCCCTTCAGCGGCCAGTAGCATCTGACTTTGAGCCTCAGGGTCTGAGTG

Translation: WPNDGEGAFHGDAEALQRPVASDFEPQGLS

(1) UCB bag #2 DNA sequence (seq5):

GGGTTRAGAMYCCAATCGAGACCTGCTGATCGATTCGAGCTCGGTCCCGGGGACCCTCAGACCCTGAAGGCTCAAAGTCAGATGCTACTGGCCGCTGAAGGGCTTCTGCGTCTCCATGGAAGGCGCCCTCGCCATCGTTGAAGCTTGGCACTGGCCGTCGTTTTACAACGTCGTGACTGGRAAAACCCTGGCGTTACCCAACTTAATCGCCTTGCAGCACATCCCCCTTTCGCCAGCTGGCGTAATAGCRAARAGGCCCGCACCGATCGCCCTTCCCAACAGTTGCGCAGCCTGAATGGCGAATGGCGCCTGATGCGGTATTTTCTCCTTACGCATCTGTGCGGTATTTCACACCGCATATGGTGCACTCTCAGTACAATCTGCTCTGATGCCGCATAGTTAAGCCAGCCCCGACACCCGCCAACACCCGYTGACGCGCCCTGACGGGCTTGTCTGCTCCCGGCATCCGYTAACARACAAGCTGTGCCCGTCTCCGGGAGCTGCATGTGTCAAATTTTTCACCGTCATCACCCAACACGCCCAAAARAAAGGSCCYCGTGATACSCCTATTTTATAGGTWATGYCWTGAWAAWARTGGTTTCTYARACYTCAGGTGSCYCTTTTCGAAAATGKGCSCGRAACCCWTTTTTTGTTTATTTTCTAATTACMTCAAAATRTGTATCCCCTCAAAAAACAAAAACCSWKAAAATTTCTTTCAATAATATTGAAAAAARGGGAATAKTTTYGAYTTTTTAMACCTTTTC

DNA alignment against BCR-ABL1 (p190):

**97.7% identity** (97.7% similar) in 87 nt overlap (140-54:7-92)

140 130 120 110 100 90

seq5 CAACGATGGCGAGGGCGCCTTCCATGGAGACGCAGAAGCCCTTCAGCGGCCAGTAGCATC

::::::::::::::::::::::::::::::::::::::::::::::::::::::::::::

p190 CAACGATGGCGAGGGCGCCTTCCATGGAGACGCAGAAGCCCTTCAGCGGCCAGTAGCATC

10 20 30 40 50 60

80 70 60

seq5 TGACTTTGAGCCT**T**CAGGGTCTGAGGG

::::::::::::: ::::::::::: :

p190 TGACTTTGAGCCT-CAGGGTCTGAGTG

70 80 90

Translated sequence alignment:

96.2% identity (100.0% similar) in 26 aa overlap (202-227:3-28)

210 220

seq5 NDGEGAFHGDAEALQRPVASDFEP**S**G

::::::::::::::::::::::::.:

p190 NDGEGAFHGDAEALQRPVASDFEPQG

10 20

(2a) UCB bag #3 DNA sequence (seq7):

GCATGATGATTACGATTCGAGCTCGGTACCCGGGGACCCTCARACCCTGAAGGCTCAAAGTCAGATGCTACTGGCCGCTGAAGGGCTTCTGCGTCTCCATGGAAGGCGCCCTCGCCATCGTTGAAGCTTGGCACTGGCCGTCGTTTTACAACGTCGTGACTGGGAAAACCCTGGCGTTACCCAACTTAATCGCCTTGCAGCACATCCCCCTTTCGCCAGCTGGCGTAATAGCGAARAGGCCCGCACCGATCGCCCTTCCCAACAGTTGCGCAGCCTGAATGGCGAATGGCGCCTGATGCGGTATTTTCTCCTTACGCATCTGTGCGGTATTTCACACCGCATATGGTGCACTCTCAGTACAATCTGCTCTGATGCCGCATAGTTAAGCCAGCCCCGACACCCGCCAACACCCGCTGACGCGCCCTGACGGGCTTGTCTGCTCCCGGCATCCGCTTACAGACAAGCTGTGACCGTCTCCGGGAGCTGCATGTGTCAGAGGTTTTCACCGTCATCACCGAAACGCGCGAGACGAAAGGGCCTCGTGATACGCCTATTTTTATAGGTTAATGTCATGATAATAATGGTTTCTTAGACGTCAGGTGGCACTTTTCGGGGAAATGTGCGCGGAACCCCTATTTGTTTATTTTTCTAAATACATTCAAATATGTATCCGCTCATGAGACAATAACCCTGATAAATGCTTCAATAATATTGAAAAAGGAAGAGTATGAGTATTCACATTTCCGTGTCSCCCTTATTCCCTTTTTTTGCGGCATTTTGCCTTCTGTTTTTGCTCCACCCAGAAACSCTGTGAAGTAAAAGATGCTGAGATCAGTGGGTGCACGAGTGGATAACATCGACTGAATCTCACAGCGTAGATCCTGAAAGTTTCGCCCGGAGACGTTTCAATGATGAGACACGTTTTAAGGTCTGC

DNA alignment against BCR-ABL1 (p190):

**96.6% identity** (97.7% similar) in 87 nt overlap (123-37:7-92)

120 110 100 90 80 70

seq7 CAACGATGGCGAGGGCGCCTTCCATGGAGACGCAGAAGCCCTTCAGCGGCCAGTAGCATC

::::::::::::::::::::::::::::::::::::::::::::::::::::::::::::

p190 CAACGATGGCGAGGGCGCCTTCCATGGAGACGCAGAAGCCCTTCAGCGGCCAGTAGCATC

10 20 30 40 50 60

60 50 40

seq7 TGACTTTGAGCCT**T**CAGGGTYTGAGGG

::::::::::::: ::::::.:::: :

p190 TGACTTTGAGCCT-CAGGGTCTGAGTG

70 80 90

Translated sequence alignment:

**96.2% identity** (100.0% similar) in 26 aa overlap (261-286:3-28)

270 280

seq7 NDGEGAFHGDAEALQRPVASDFEP**S**G

::::::::::::::::::::::::.:

p190 NDGEGAFHGDAEALQRPVASDFEPQG

10 20

(2b) UCB bag #3 DNA sequence (seq10):

CCCTCATGATTACGATTCGAGCTCGGTACCCGGGGACCCTCARACCCTGAAGGCTCAAAGTCAGATGCTACTGGCCGCTGAAGGGCTTCTGCGTCTCCATGGAAGGCGCCCTCGCCATCGTTGAAGCTTGGCACTGGCCGTCGTTTTACAACGTCGTGACTGGGAAAACCCTGGCGTTACCCAACTTAATCGCCTTGCAGCACATCCCCCTTTCGCCAGCTGGCGTAATAGCGAARAGGCCCGCACCGATCGCCCTTCCCAACAGTTGCGCAGCCTGAATGGCGAATGGCGCCTGATGCGGTATTTTCTCCTTACGCATCTGTGCGGTATTTCACACCGCATATGGTGCACTCTCAGTACAATCTGCTCTGATGCCGCATAGTTAAGCCAGCCCCGACACCCGCCAACACCCGCTGACGCGCCCTGACGGGCTTGTCTGCTCCCGGCATCCGCTTACAGACAAGCTGTGACCGTCTCCGGGAGCTGCATGTGTCAGAGGTTTTCMCCGTCATCACCGAAAACGCGCGAGAMGAAAGGGCCTCGTGATACSCTAATTTTTATAGGTTAATGTCATGATAATAATGGTWTCTTAGACSGTCRGGKGGCACTTTTTCGGGGRAAATGTGCCGCGGAAACCCCCTWTTTTGGTTTWTTTTTTCTAAAATACATTCAAATATGTATCCGCTYCATGAGACAAWTAAMCCCTGAATAAAATGCTTCATWATTATTGGCAAATGGAGAGTATGAGTATTCCACATTTCCGTGTCGTCCATAATCCCCATTATGGCGCATTTTGGCCATACTGGATATTAGCTCAAACGGRGACCACTGTGGTAGGGTCACAGGTCTAGAAATTTCTGCCTTGTTKKCCACC

96.6% identity (97.7% similar) in 87 nt overlap (123-37:7-92)

120 110 100 90 80 70

seq10 CAACGATGGCGAGGGCGCCTTCCATGGAGACGCAGAAGCCCTTCAGCGGCCAGTAGCATC

::::::::::::::::::::::::::::::::::::::::::::::::::::::::::::

p190 CAACGATGGCGAGGGCGCCTTCCATGGAGACGCAGAAGCCCTTCAGCGGCCAGTAGCATC

10 20 30 40 50 60

60 50 40

seq10 TGACTTTGAGCCT**T**CAGGGTYTGAGGG

::::::::::::: ::::::.:::: :

p190 TGACTTTGAGCCT-CAGGGTCTGAGTG

70 80 90

Translated sequence alignment:

96.2% identity (100.0% similar) in 26 aa overlap (244-269:3-28)

250 260

seq10 NDGEGAFHGDAEALQRPVASDFEP**S**G

::::::::::::::::::::::::.:

p190 NDGEGAFHGDAEALQRPVASDFEPQG

10 20

Note: Extra **T**-residue in the sequence of BCR-ABL PFG of probands (1) seq5, (2a) seq7, and (2b) seq10, and resulting assumed Q → S substitution in probands’ BCR-ABL fusion protein was introduced with ABL-reverse primer containing +T that was used for amplification of qPCR product by standard PCR (for subcloning of amplified qPCR product into sequencing vector, see Materials and Methods, section Sequencing of R-T qPCR products)

**Gabert’s** MLL-AF4 sequence

GATGGAGTCCACAGGATCAGAGTGGACTTTAAGCAGACCTACTCCAATGAAGTCCATTGTGTTGAAGAGATTCTGAAGGAAATGACCCATTCATGGCCGCCTCCTTTGACAGCAATACATACGCCTAGTACAGCTGAGCCATCCAAGTTTCCTTTC

**Translation:** DGVHRIRVDFKQTYSNEVHCVEEILKEMTHSWPPPLTAIHTPSTAEPSKFPF

(3) UCB bag #2 DNA sequence (seq10):

GWCTAAMYYMTATCTGTCCTGCTGATMGATTCGGACTTGGWGGCTCAGCTGTMTAGGCGTATGTATTGCTGTCAAAGGAGGCGGCCATGAATGGGTCATTTCCTTCAGAATCTCTTCAACACAATGGACTTCATTGGAGTAGGTCTGCTTAAAGTCCACTCTGATCCTGTGGACTCCAAGCTTGGCACTGGCCGTCKTTTTACAACGTCGTGACTGGGAAAACCCTGGCGTTACCCAACTTAATCGCCTTGCAGCACATCCCCCTTTCGCCAGCTGGCGTAATAGCGAARAGGCCCGCACCGATCGCCCTTCCCAACAGTTGCSCASCCTGAATGGCGAATGGCSCCTGATGCGGWATTTTCTCCTTACGCATCTGGTGCGGTATTTCACACCGSMATATGGKGCACTTCTCAGTACAATCTGCTTCTGATGCCSCATARTTAAYCTTTCCCCCRAACACCCCSCCAACACCCCGYTGAAGSCGCCCTGACGGGGYTTGTCTGGCTCCCGGGCAATCCGGTTGGGGGGGAGGGGGKKGKGAMCCCYCCCCCCCCSMAGYTGGMWTGTTGYCCRRAAGGGTTTTTCCCCCGMCCAYCCMCCRAAAACCGCGKGRAAAAMRAAARGGGCCCCTCSKGAAAWACCCCWAATTTTTTTAWKGGGGTWAAWGGYCMRKGGATAAATAAAGGGGTTTCCTWAAAASCYCAGGGGGGGCAYCTTTTTTCGGGGGAAAAWTKGKSCCACSRRAAMCYCCCTYTWTTTTTGAWTYTATATTTTTCTRARAAAAWCASTTTC

DNA alignment against MLL-AF4

97.3% identity (98.6% similar) in 146 nt overlap (178-35:3-148)

170 160 150 140 130 120

seq2 TGGAGTCCACAGGATCAGAGTGGACTTTAAGCAGACCTACTCCAATGAAGTCCATTGTGT

::::::::::::::::::::::::::::::::::::::::::::::::::::::::::::

MLL-AF4 TGGAGTCCACAGGATCAGAGTGGACTTTAAGCAGACCTACTCCAATGAAGTCCATTGTGT

10 20 30 40 50 60

110 100 90 80 70 60

seq2 TGAAGAGATTCTGAAGGAAATGACCCATTCATGGCCGCCTCCTTTGACAGCAATACATAC

::::::::::::::::::::::::::::::::::::::::::::::::::::::::::::

MLL-AF4 TGAAGAGATTCTGAAGGAAATGACCCATTCATGGCCGCCTCCTTTGACAGCAATACATAC

70 80 90 100 110 120

50 40

seq2 GCCTAK-ACAGCTGAGCCW-CCAAGT

:::::. :::::::::::. ::::::

MLL-AF4 GCCTAGTACAGCTGAGCCATCCAAGT

1. 140

Translated sequence alignment:

**100.0% identity** (100.0% similar) in 41 aa overlap (206-246:2-42)

210 220 230 240

seq2 GVHRIRVDFKQTYSNEVHCVEEILKEMTHSWPPPLTAIHTP

:::::::::::::::::::::::::::::::::::::::::

MLL-AF4 GVHRIRVDFKQTYSNEVHCVEEILKEMTHSWPPPLTAIHTP

10 20 30 40
